# Complement as a regulator of adaptive immunity

**DOI:** 10.1007/s00281-017-0644-y

**Published:** 2017-08-25

**Authors:** Justin Killick, Gregoire Morisse, Dirk Sieger, Anne L. Astier

**Affiliations:** 1 MRC Centre for Inflammation Research, Edinburgh Centre for MS Research, University of Edinburgh, Queen’s Medical Research Institute, Edinburgh, EH16 4TJ UK; 2 Centre for NeuroRegeneration, Edinburgh Centre for MS Research, University of Edinburgh, Edinburgh, EH16 4SB UK; 3 Inserm U1043, CNRS U5282, Université de Toulouse, Centre de Physiopathologie Toulouse-Purpan (CPTP), F-31300 Toulouse, France

**Keywords:** Complement, Adaptive immunity, T cells, B cells, CD46, Inflammation

## Abstract

The complement system is an ancient and evolutionarily conserved effector system comprising in mammals over 50 circulating and membrane bound proteins. Complement has long been described as belonging to the innate immune system; however, a number of recent studies have demonstrated its key role in the modulation of the adaptive immune response. This review does not set out to be an exhaustive list of the numerous interactions of the many complement components with adaptive immunity; rather, we will focus more precisely on the role of some complement molecules in the regulation of antigen presenting cells, as well as on their direct effect on the activation of the core adaptive immune cells, B and T lymphocytes. Recent reports on the local production and activation of complement proteins also suggest a major role in the control of effector responses. The crucial role of complement in adaptive immunity is further highlighted by several examples of dysregulation of these pathways in human diseases.

## Introduction

For many years, the complement system has been described as being a key element in innate immunity. This ancient evolutionary system is indeed crucial to fight pathogens, by patrolling as sentinels in the circulation and targeting these invaders for destruction, as well as initiating inflammatory responses [1]. In the past decade, it has become clear that components of the complement system also represent an integral part of the regulation of the adaptive immune response. Indeed, complement components and their receptors are expressed and produced by adaptive immune cells. The interaction of complement proteins and their cognate receptors has been shown to control several functions ranging from activation, differentiation, and metabolism of adaptive immune cells [2]. The recent discovery that complement is not only present in serum and other fluids but also has been shown to be activated intracellularly, including in lymphocytes, opens novel doors on the role of the complex interactions between this innate system and the control of adaptive immune responses [3]. This review will present recent advances in this field, with a particular focus on the regulation of B and T cell activation by complement components, and how dysregulation of these controls can lead to pathogenesis.

### Adaptive immunity

Immune responses have traditionally been grouped into either innate or adaptive responses. The innate response is fast but non-specific. The complement system is one of the first parts of the innate response to be activated when “foreigners” such as bacteria invade the body, and it presents an immediate and critical tool to help defend against infections and clear pathogens by allowing their opsonization and further killing [1]. Adaptive immunity on the other hand, although slower, elicits a very specific immune response through the activation of immune cells (T cells and B cells) that express highly specific antigen receptors. The adaptive immune response is also able to develop a memory to the antigen, allowing a faster and stronger response upon re-encounter. In recent years, a finely tuned interplay between these two types of immune response has been reported and is now extensively studied. Notably, the complement system has been shown to directly control B and T cell responses [2, 4].

Naïve T cells are activated by their cognate TCR following the recognition of a specific peptide from a given antigen presented by the MHC, hence conferring the specificity of the T cell response. Full activation, however, requires co-stimulation and is provided by the engagement of co-stimulatory molecules expressed on T cells and ligated by their cognate receptors at the surface of antigen presenting cells (APC) [5]. Depending on the cytokines and signals received by the naïve T cells, these will differentiate within different T helper (Th) subsets [6]. These differentiated Th subsets are, however, not static as they exhibit a certain level of plasticity and are able to modulate their cytokine profiles depending on the surrounding environment and the signals received. In counterpoint to these effector Th subsets, a variety of regulatory T cells (Tregs) operate, essential to control immune homeostasis by counteracting overactivation of effector T cells [7]. Lack of regulatory T cells or dysfunction of these cells is indeed detrimental to health, giving rise to allergy or autoimmune diseases.

Similarly, B cells are adaptive immune cells that are specific to a given antigen. As opposed to T cells, B cells recognize the whole antigen and not a processed peptide. Recognition of the antigen is made by the unique B cell receptor (BCR) expressed at the cell surface, namely a membrane-bound immunoglobulin. Full activation of B cells also requires co-stimulation alongside BCR stimulation, such as ligation of the cell surface molecule CD40. Recent studies have highlighted the existence of specific regulatory B cells (Bregs) that also contribute to the maintenance of immune homeostasis in a manner analogous to Tregs [8].

### Complement components and their receptors and regulators modulating adaptive immunity

This review does not expand on the different complement activation pathways and cascades leading to the formation of membrane attack complexes (MAC) as this is covered in depth in other reviews in this issue. We will, however, briefly describe some of the complement receptors and complement regulators that are involved in the regulation of the adaptive immune cells, as further discussed below.

Complement activation results in the generation of C3 and C5 convertase complexes; these will cleave C3 and C5, respectively, generating the anaphylatoxin components C3a and C5a as well as the opsonin C3b and MAC initiator C5b. Binding of C3 and C5 fragments to their cognate receptors/regulators triggers intracellular signaling resulting in the modulation of key cellular functions.

#### Complement receptors

CD21, also called complement receptor 2 (CR2), is a cell surface glycoprotein which binds and recognizes a variety of ligands, such as the Epstein-Barr virus, the IgE receptor CD23, IFNα, and the complement C3 degradation products C3d, iC3b, and C3d,g. CD21 profoundly modulates B cell functions, as further discussed below.

Complement C3a receptor, C3aR, is a G-protein-coupled receptor (GPCR) which interacts with complement component C3a. C3aR has been shown to be present on both B cells and T cells; however, much of the literature has focused on its role in modulating T cell-mediated immunity [9]. In resting T cells, C3aR is only expressed intracellularly within lysosomes, while upon activation, the receptor is translocated to the plasma membrane [3].

Complement C5a receptor 1, C5aR1 (CD88), is a GPCR which binds to the complement components C5a and desarginated C5a (C5a^desArg^). Although initially suggested that C5a^desArg^ bound to C5aR1 with less affinity than C5a [10], this was recently challenged [11]. Its localization appears to be somewhat dependent on cell type, as it is detected both in the cytoplasm and on cell membrane in monocytes whereas it appears to be exclusively localized within the cell in T cells [12, 13]. C5aR1 is present on a variety of cell types, not exclusively immune cells. Its interaction with C5a is believed to be pro-inflammatory and contributes to the pathogenesis of several inflammatory diseases.

C5aR2 (or C5L2) binds to both C5a and C5a^desArg^ with comparable affinity to C5aR1. C5aR2 is a seven-transmembrane domain receptor, localized primarily intracellularly but also expressed on the cell membrane [12]. C5aR2 shares 38% sequence homology with the C5aR1 receptor but does not couple to G proteins [14]. The lack of GPCR-coupled signaling led to the belief that this receptor was a “decoy” receptor with no other function than to compete for C5a binding with C5aR1 [15]. However, recent studies have shown that C5aR2 can recruit and signal through β-arrestin, having both pro- and anti-inflammatory effects depending on the cell type and signaling conditions [13].

#### Complement regulators

CD46, also known as membrane co-factor protein (MCP), is a member of the regulators of complement activation (RCA) family. CD46 is ubiquitously expressed (except for erythrocytes) and, along with other complement regulators, protects cells from autologous complement mediated lysis, by binding to complement components C3b and C4b and facilitating their degradation by factor I. CD46 also acts as a receptor for several pathogens including measles and adenoviruses and the *Neisseria (N) gonorrhoeae* and *N meningitides* bacteria [16]. In addition, CD46 is a powerful regulator of T cell-mediated immunity, as further discussed below.

CD55, also known as decay accelerating factor (DAF), is a glycosylphosphatidylinositol (GPI)-anchored cell surface molecule, and a member of the RCA family. CD55 promotes the degradation and inhibits the formation of complement C3 and C5 convertases and thus prevents amplification of the complement cascade and formation of the MAC.

CD59, another GPI-anchored molecule, prevents complement-mediated lysis of autologous cells by inhibiting the interaction between complement C9 and C5b-8 complex, hence preventing the formation of the MAC [17].

CD35, or complement receptor 1 (CR1), is a transmembrane glycoprotein and a member of the RCA family. CD35 binds the ligands C3b, iC3b, and C4b. Like CD55, CD35 has decay accelerating activity promoting the degradation of complement C3 and C5 convertases. However, unlike other members of the RCA family, CD35 possesses both decay accelerating activity and cofactor activity for factor I-mediated complement cleavage. CD35 catalyzes factor I cleavage of iC3b to C3c and C3dg, the latter being a ligand for CD21 [18].

C4b binding protein (C4BP) is a multimeric serum soluble glycoprotein produced and secreted primarily by the liver. Several isoforms of C4BP exist, composed of various combinations of alpha and beta chains. C4BP has both decay accelerating activity and cofactor activity for factor I-mediated cleavage, resulting in the dissociation of C3 convertases and degradation of C3b and C4b, respectively. Serum localized C4BP forms a complex with vitamin-K-dependent protein S, which allows binding to negatively charged phospholipids such as the apoptotic cell marker phosphatidylserine [19]. The binding of C4BP to apoptotic cells inhibits complement C3 and C5 convertase formation and subsequent lysis by MAC formation, preventing the induction of an inflammatory response due to excessive complement activation and the release of cellular contents due to cell lysis [20].

Factor H (FH) is a soluble complement regulator present in the plasma [21]. It binds and inhibits C3b. Factor H acts as a co-factor for factor I-mediated cleavage of complement component C3b to iC3b, preventing the assembly of the C3bBb alternative pathway C3 convertase. Factor H can also facilitate the decay of already formed C3bBb C3-convertase by displacing bound Bb from C3b.

### Complement in APC function

One of the primary functions of the innate immune system is the recognition, uptake, and presentation of foreign pathogens to activate the adaptive immune system. Upon recognition of an antigen by APC, such as dendritic cells (DCs), the entity is engulfed, digested, and the subsequent antigenic peptide is presented on MHC receptors at the APC surface to activate the specific T cells. The serum complement system forms an integral part of this process through the opsonization of foreign entities, which improves antigen recognition and uptake into APCs via complement receptors CD21 and CD35 [22]. DCs, along with macrophages and mast cells, are one of the largest producers of extra-hepatic C1q which induces cellular responses on local tissues in a paracrine manner [23]. C1q induces maturation of DCs and upregulates expression of cell surface MHC class II and CCR7, the latter being a chemokine receptor necessary for DC migration towards the lymphoid tissue [24]. C1q-matured DCs also secreted higher amounts of IL-12p70 which in turn stimulates a greater Th1 response from co-cultured T cells [24]. However, C1q bound to apoptotic cells induced DCs to secrete IL-10 as opposed to IL-12p70, suppressing Th1 and Th17 cell proliferation [25]. DC production of C1q ceases upon maturation, which may represent a negative feedback loop, limiting DC maturation; it may also serve to restrict C1q production in lymphoid tissues where it could have a direct impact on B and T cell responses [23].

In a model of influenza infection, C3 is required for the migration of lung DCs to the lymph nodes [26]. CD46 ligation by measles virus or antibodies on human DCs has been reported to modulate secretion of the pro-inflammatory cytokines IL-12 and/or IL-23 [27–29]. Hence, complement modulates the ability of DCs to migrate towards the lymphoid tissue and modulates the adaptive response through regulation of cytokine secretion. Local production of C3a and C5a at the APC–T cell interface is also key to regulate T cell activation and survival [30]. Exogenous FH also modulates the maturation and function of DCs and their ability to stimulate T cells. Treatment of monocyte-derived DC (MoDC) with FH prior to LPS stimulation resulted in the generation of a phenotypically immature MoDC population. These cells displayed reduced antigen uptake, CCR7 expression, and chemotactic ability. When co-cultured with CD3+ T cells, they induced a CD4^+^CD127^low^CD25^high^Foxp3^+^ regulatory T cell population [31]. In concordance with these observations, it was shown that IFNγ-activated DCs resulted in increased expression of cell surface factor H. Inhibition of factor H expression resulted in increased CD4+ T cell activation and proliferation. These findings suggest a role for factor H in the regulation of DC function and its modulation of T cell responses [32].

### Role of complement in B cell regulation

Complement plays a modulatory role in a variety of B cell functions, including activation and differentiation, antigen internalization and presentation, and immunoglobulin class switching. The importance of these interactions is highlighted by the variety of diseases that can occur when the complement system becomes dysregulated.

#### B cell activation

Naive B cells require two activation signals to become optimally activated, begin proliferating and generating antigen specific antibodies. The first activation signal is propagated through the stimulation of the BCR and its co-receptor complex composed of complement receptor 2 (CR2, CD21)/CD19/CD81 [33]. The second activation signal typically involves CD40 on B cells and its cognate ligand on T cells, CD154 (CD40L) [34]. Co-engagement of the BCR and of CD21 by C3d-opsoninized antigen enhances B cell activation [4, 35], by reducing the amount of antigen required for activation by between two- and four-fold; thus, C3d has been described as an antigen adjuvant. Co-engagement of the BCR and CD21/CR2 is also key to the generation of B cell memory [4].

B cell surface complement receptor 1 (CD35, CR1) binds to the complement components C3b and C4b, acting as a co-factor to facilitate their cleavage by factor I to iC3b, a substrate for CD21, and iC4b, respectively [36]. While CD21, as mentioned previously, promotes B cell activation, CD35 has an antagonistic effect, suppressing B cell activation and proliferation [37].

#### Antigen internalization and presentation

The interaction of C3 fragments with CD21 plays a fundamental role in B cell antigen internalization and presentation [38]. Internalized antigen is processed and the peptide presented on surface MHC-Cl.II receptors to activate local T cell responses [39]. Brimnes et al. [40] observed that B cells incubated in either serum-free media or with heat or chemically inactivated complement showed significantly reduced ability to uptake antigen. Blockage of CD21 with polyclonal antibodies also significantly reduced antigen uptake and presentation. The B cell uptake of complement C3d-coated antigen plays an important role in formation and maintenance of germinal centers and the subsequent differentiation of memory and effector B cells. B cells are observed to transfer complement-coated antigen to follicular DCs via the CR2 receptor; these store the antigen, periodically transferring it back to the B cells to maintain the germinal centers and extend the humoral response [22, 41].

#### Immunoglobulin class switching

**Fig. 1:**
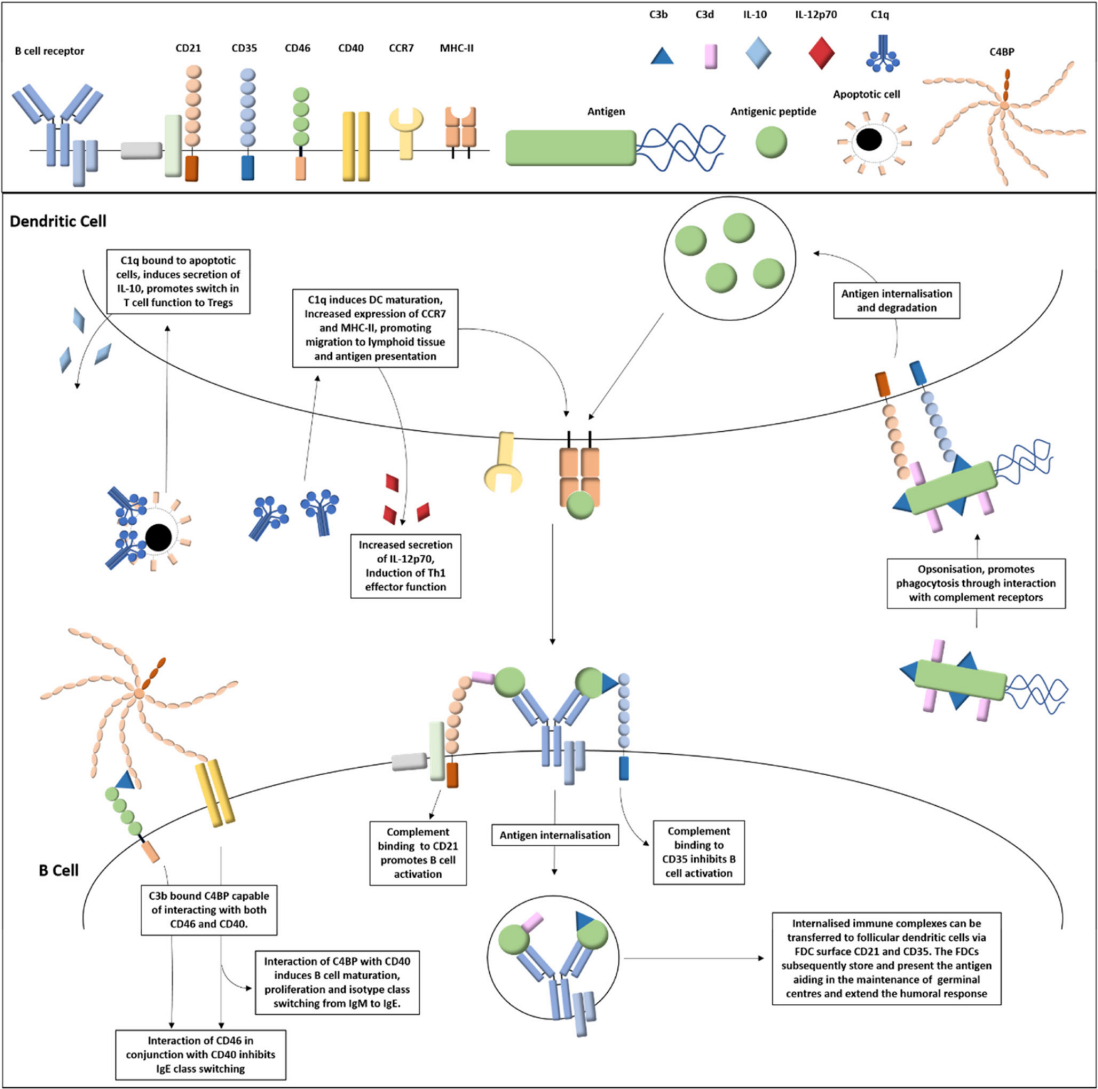
Complement regulation of dendritic and B cell functions. The interaction of complement component C1q with dendritic C1qR results in differential responses dependent on its ligand. C1q bound to microbial surfaces results in DC maturation, increased expression of MHC-II and CCR7, and increased secretion of IL-12p70 resulting in improved ability to present antigen, migration towards the lymphoid tissue and promotion of Th1 effector cell differentiation, respectively. However, C1q bound to apoptotic cells results in secretion of the anti-inflammatory cytokine IL-10 as opposed to IL-12p70 resulting in activation of regulatory T cells. C3b and C3d complement coated antigen is bound by antigen presenting cell surface CD21 and CD35 complement receptors, assisting in antigen uptake. The internalized antigen is degraded to antigenic peptide and presented on the surface MHC-II. B cell activation is regulated by complement C3d or C3b binding to cell surface CD21 or CD35 resulting in either a reduction in activation threshold or an inhibition of activation, respectively. Complement component C4BP interacts with a variety of ligands including complement C3b and B cell surface CD40. Interaction of C4BP with CD40 alone induces B cell activation, proliferation, and antibody class switching from IgM to IgE. However, C3b bound C4BP can bridge interactions between CD46 and CD40 which results in inhibition of CD40 mediated antibody class switching

The complement receptor CD21 and the complement regulators CD46 and C4BP modulate B cell immunoglobulin class switching, particularly IgE switching. Resting B cells typically express the IgM or IgD immunoglobulin isotypes. Upon differentiation, B cells switch their class immunoglobulins depending on surrounding cytokines and interaction with cell surface receptors such CD40 and the IgE receptor CD23. The earliest indication of the involvement of CD21 in IgE class switching was the discovery of its ability to bind to CD23 [42]. CD21 bound to either soluble CD23, Epstein-Barr virus fragments or monoclonal antibodies in the presence of the stimulatory signals IL-4 and anti-CD40, results in an increased production of mature IgE mRNA and secreted IgE [42, 43]. Geha’s group reported that the complement regulator C4BP binds to B cell surface CD40 inducing B cell activation and proliferation, and, in the presence of IL-4, also induces immunoglobulin class switching to promote the secretion of IgE [44]. Although CD46 stimulation has been observed to increase IgE germline transcripts, this does not result in increased secretion of IgE [45]. Moreover, co-ligation of CD46 with CD40 on B cells inhibits IgE class switching through blockage of CD40-mediated NFκB activation. Hence, a variety of complement components can differentially modulate IgE secretion depending on the target receptors involved. A summary of the roles of complement in antigen presentation and B cell function is depicted in Fig. 1.

### Role of complement in T cell activation

A growing body of evidence suggests that several members of the complement family interact with CD4+ T cells through interaction with cell surface and intracellular receptors modulating activation and subset differentiation.

#### T cell activation and differentiation

Activation by CD46 has been shown to profoundly affect T cell activation. Co-stimulation by CD3/CD46 transduces signals resulting in a strong proliferative response of activated T cells [46–48], and differentiation into Th1 cells characterized by IFNγ production [49]. As IL-2 accumulates in the surroundings of the cells, a contraction phase occurs, with decreased IFNγ production but enhanced secretion of IL-10. The release of high levels of IL-10 and low IFNγ leads to suppression of proliferation of bystander T cells; hence, CD46-costimulated T cells acquire a phenotype of so-called type I regulatory T cells (Tr1) [50]. CD46 co-stimulation modulates expression of Notch family members at the surface of activated T cells, while on resting T cells, CD46 binds to the Notch family member Jagged1, inhibiting both Notch signaling and CD46 activation. Activation of CD46 leads to CD46 downregulation, freeing Jagged 1 to interact with Notch [51]. Interestingly, Notch regulates IL-10 secretion in Th1 cells [52]. Hence, CD46 may provide the link between the Notch and complement cascades to regulate T cell differentiation and IL-10 production.

Enzymatic processing of CD46 occurs upon T cell activation, leading to the shedding of its extracellular domain and subsequent cleavage of its two cytoplasmic tails, Cyt1 and Cyt2. The two tails exhibit antagonistic roles in the control of inflammation, in both huCD46-transgenic mice (mice only express CD46 on testis) [53] and in human T cells in vitro [54, 55]. Processing of CD46 is required for IL-10 production and allows T cell activation but also T cell termination [54, 55]. Both tails contain a nuclear localization signal (NLS) sequence, and indeed, cleaved tails translocate to the nucleus likely controlling target genes [56]. CD46 expression on activated T cells is tightly controlled [57–60]. Vitamin D, for example, which is known to promote immune regulation, triggers the Th1 to Tr1 switch and favors CD46 cleavage [60]. Expression of CD46 on activated T cells is also under control of a crosstalk with CD28 [61]. Regulation of the splicing of the Cyt1 exon have been recently reported [62]. A few interactors of CD46 tails (albeit only with Cyt1) have been identified, leading to the discovery of the role of CD46 in controlling autophagy (via interaction with GOPC) [63] and cell polarity via binding to DLG4 [64, 65]. Cyt1 interacts with the kinase SPAK, and this is required for IL-10 production [49]. CD46 also interacts with alpha-E-catenin in human CD4+ T cells and knockdown of α-E-catenin impaired CD46 downregulation suggesting a role for α-E-catenin in CD46-controlled expression [51]. CD46 is recruited to lipid rafts [66] and T cell polarization is affected by CD46 ligation [47, 65]. CD46 ligation before specific T cell activation can prevent subsequent TCR signaling by recruiting the lipid rafts and preventing the immune synapse formation [65], while co-ligation with the TCR promotes its recruitment to the immune synapse (S. Ni Choileain, J. Hay, et al., submitted). Together, these studies highlight the key role of CD46 signaling on controlling T cell differentiation and function.

Other members of the RCA family also control T cell activation. Similar to CD46, CD55 affects co-stimulation of human T cells by binding to CD97, promoting T cell activation and Tr1 cell differentiation [67]. In mice, it was observed that the lack of CD55 promotes T cell activation, suggesting an inhibitory role of CD55 in T cell effector function [68, 69]. A role for CD59 in T cell co-stimulation has also been reported [70] and shown to couple signaling events to the TCR [71]. Moreover, recent studies have highlighted an immunomodulatory role of CD59 in T cells suggesting that its ligation may also potentially induce a regulatory T cell subset [72].

Not only complement regulators but also complement receptors modulate T cell function and survival. In mice, C5aR and C3aR act as costimulatory signals for T cells and sustain naive T cell survival [30]. C3aR is not expressed at the cell surface of naïve T cells but on lysosomes and is transported to the surface upon T cell activation [73]. Defective cytokine secretion by T cells from *C3ar1* ^−/−^ and *C5ar1* ^−/−^ mice show that these receptors are also needed for effector function [30]. In addition, these complement receptors control Treg function. nTregs express C3aR and C5aR, and triggering of these receptors inhibits Treg suppressive function by modulating Foxp3 expression [74]. *C3aR* ^*−/−*^ and *C5aR* ^*−/−*^ mice have increased levels of Foxp3 + Tregs [75]. Similarly, blocking these receptors with C3aR and C5aR antagonists in human T cells induced suppressive human iTregs [75].

#### Production of local and intracellular C3 and C5 in T cells

Until recently, it was thought that soluble complement components were mostly present in the plasma after production mainly by the liver. In recent years, a series of papers have demonstrated the local production of C3 and C5 fragments by adaptive immune cells. In mice, T cell-derived C3 activation is due to the formation of C3 convertase [30]. In contrast, in resting human T cells, a recent report suggests that there is continuous production of C3 that is cleaved by cathepsin L (CTSL), giving rise to C3a and C3b [3]. The resulting C3a binds to its C3aR at the surface of lysosomes and participates to T cell survival by regulation of mechanistic target of rapamycin (mTOR) activity [3]. Upon TCR activation, both C3a and C3b are exported to the cell surface within minutes and bind to surface C3aR, also expressed at the surface after activation, and CD46, respectively, leading to T cell activation and Th1 differentiation. Uncontrolled cleavage results in hyperactivation of Th1 responses, such as that observed in a small number of patients with rheumatoid arthritis, and indeed, inhibition of CTSL normalized the response [3].

Interestingly, CTSL has been linked to inflammatory responses and IL-17 production by controlling Th17 differentiation in both humans and mice [76, 77], suggesting a broader role of cathepsins in regulating inflammatory conditions. This also suggests a role of the C3 and C5 fragments in modulating Th17 response.

The same group recently reported the intracellular cleavage of C5 in T cells upon co-stimulation by CD3/CD46 [12]. Binding of cleaved C5a to intracellular C5R1 leads to activation of the NLRP3 inflammasome in T cells, and IL-1β production has been reported [12]. The NLRP3-IL-1β axis participates in the activation of CD46 in T cells and IFNγ production. On the other hand, binding of C5a to C5aR2 that is expressed at the cell surface inhibits this pathway [12]. Of note, we have failed to detect any IL-1β secreted upon CD46 costimulation of T cells from either healthy donors or patients with multiple sclerosis (A. Itchers, J. Killick, A. Astier, unpublished data), and the reasons for these discrepancies are unknown and may relate to the sensitivity of the assays used. Further research will allow clarification on the role of this pathway in T cell differentiation.

It is important to note that key differences between men and mice have been reported. Local production of C3 and C5 fragments has been demonstrated in mice, although this was thought to be due to increased C3 expression and extracellular cleavage by the C3 convertase [78]. Moreover, although CTSL and C3 are present in murine T cells, normal Th1 differentiation occurred in T cells from CTSL knockout mice, suggesting that C3 fragments do not require CTSL processing in murine T cells, as opposed to human cells [3]. Moreover, mice do not express CD46 (except for testis), implying that the interaction between C3b and CD46 is not required to generate a Th1 response. It would be interesting to assess the role of the C3b receptor and complement regulator Crry in this response, especially as Crry has been also shown to act as a costimulatory molecule for murine T cells [79], similarly to CD46 engagement on human T cells [46].

Importantly, although the role of intracellular complement activation has so far been only shown in human CD4 T cells, intracellular complement activation has been observed in several cell types by the authors, suggesting that this is a general biologic process necessary to overall cell function [3], and the term “complosome” has recently been suggested [80].

### Role of complement in T cell metabolism

Resting T cells typically have low energy requirements needed to maintain homeostatic functioning. As such, they require only a limited influx of nutrients and have low glycolytic activity, instead deriving energy through mitochondrial oxidative phosphorylation. Upon activation, the energy requirements of the cells rise to support the increased energy demands of cellular proliferation and effector functions [81, 82]. To meet this demand, the activated cells enhance generation of ATP through both glycolysis and oxidative phosphorylation pathways. This process of metabolic adaption to meet the increased energy demands is referred to as metabolic reprogramming. Upon activation, T cells upregulate surface expression of the glucose channel GLUT1 [83] and the amino acid channel LAT1 [84] resulting in increased influx of nutrients. This increased nutrient uptake, particularly the augmented influx of amino acids through the LAT1 channel, is sensed by mTOR [85, 86]. This subsequently enhances the expression of the transcription factors myc and HIF1α, resulting in increased expression of the glycolytic machinery required for metabolic reprogramming and effector function [85, 87]. While a limitation of glucose availability severely impacts T cell activation and effector function [84, 87, 88], T cells display improved effector function with increased glucose [88].

**Fig. 2:**
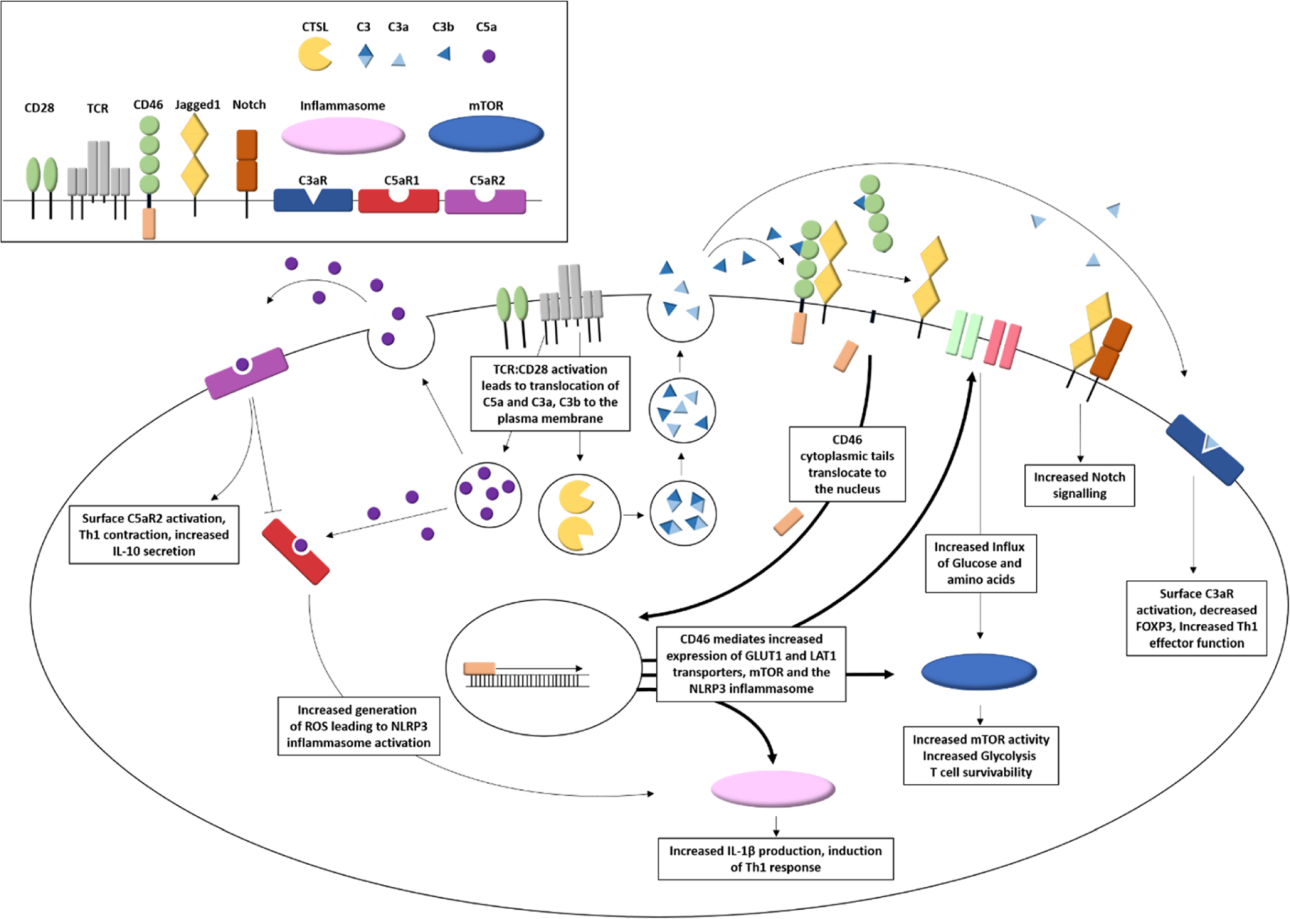
T cell activation induces autocrine and intracellular complement activation that regulates T cell metabolic function and inflammasome formation. T cell activation by TCR/CD28 results in the intracellular generation of C5a and the cathepsin L-mediated cleavage of intracellular C3 to C3a and C3b. Intracellular C5a activates cytosolic C5aR1, in addition to being translocated to the plasma membrane allowing interaction with the cell surface C5aR2. Activation of C5aR1 leads to an increase in intracellular ROS generation, which in turn results in the formation of the NLRP3 inflammasome complex. Activation of C5aR2 by cell secreted C5a negatively regulates this process. Lysosomes containing the C3 cleavage products C3a and C3b are shuttled to the cell membrane leading to C3a and C3b mediated autocrine activation of C3aR and CD46, respectively. Activation of cell surface C3aR results in decreased expression of the regulatory T cell transcription factor FOXP3 and increased Th1 effector functions and IFNγ secretion. Cell surface CD46 forms a complex the Notch signaling activator Jagged1. Upon interaction of C3b to CD46, CD46 extracellular region is cleaved by metalloproteases and its intracellular cytoplasmic domains further cleaved by a presenilin-gamma secretase complex. Cleavage releases Jagged1 resulting in activation of the Notch signaling pathway. Translocation of the CD46 cytoplasmic tail to the nucleus results in increased expression of GLUT1 and LAT1 nutrient transporters, mTOR, and the NLRP3 inflammasome

As discussed earlier, upon TCR activation intracellular stores of C3a and C3b are translocated to the cell surface where they interact with cell surface C3aR and CD46, respectively [30, 49]. The interaction between C3b and CD46 modulates the metabolic reprogramming dependent on the CD46 cytoplasmic tails [56]. CD46–Cyt1 isoform when bound by C3b signal an increase in cell surface expression of the glucose channel, GLUT1 and the amino acid channel, LAT1, by modulation of miR-150 expression [89]. Moreover, an increase in the expression of MAPK and the mTOR activator 5 (LAMTOR5), which resulted in increased mTOR assembly and glycolysis, was observed. In contrast, activation of the CD46–Cyt2 isoform resulted in a decrease in the rate of glycolysis towards levels observed prior to activation, resulting in Th1 contraction and increased secretion of IL-10. Hence, CD46, depending on its cytoplasmic domain, may lead to increased metabolic state supporting proliferation and effector function of activated T cells before mediating a switch to a lower glycolytic profile encouraging Th1 contraction and generation of suppressive IL-10 secreting T cells. The key role of CD46 in T cell activation is summarized in Fig. 2. Of note, enzymatic processing of the tails is required to control T cell activation and cytokine production. While cleavage of Cyt1 is required to promote IL-10 production, cleavage of Cyt2 was shown to decrease IFNγ production and reduce overall T cell function [54, 55]. Hence, it is clear that the balanced expression of the tails profoundly governs T cell function, and a deeper understanding of the mechanisms regulating their expression and function is needed.

### Aberrant complement regulation in human diseases

The importance of complement in controlling immune homeostasis is highlighted by the dysregulation of these pathways in a large number of diseases. Analysis of the circulating complement products may also act as biomarkers, as for example increased levels of complement components offer a differential diagnosis between MS and the relatively close neuromyelitis optica spectrum disorder [90]. As discussed earlier, CD46 is key in regulating T cell function. The CD46 pathway has been shown to be dysfunctional in a number of chronic inflammatory diseases. An impaired production of IL-10 upon CD46 co-stimulation was observed in patients with relapsing-remitting multiple sclerosis (MS). This was specific to CD46 costimulation as levels of IL-10 were normal when T cells were activated by CD28, indicating a specific defect in the CD46 pathway [57, 91, 92]. As mice do not express CD46 on somatic cells, the role of CD46 has been studied using an MS model in monkeys that express CD46 on every cell type in a similar fashion to man (although unlike humans, non-human primates also express CD46 on red blood cells), and a similar dysfunction of the regulatory function of CD46 on T cells was observed [93]. The lack of regulatory Th1-Tr1 switch was also reported in rheumatoid arthritis [49] as well as asthma patients [94, 95]. Hence, chronic inflammation switches CD46 towards an inflammatory response. This was also observed for DCs in MS as CD46-activated DCs produce increased levels of pro-inflammatory IL-23 and chemokines compared to healthy DCs [29]. The exact molecular mechanisms responsible for this inflammatory switch during inflammation are not totally understood and warrant further research. T cell activation leads to a change in glycosylation of CD46 that is required for correct signaling and processing. This regulatory mechanism appears to be altered in T cells from patients with MS, leading to a dysfunctional pathway (S. Ni Choileain, J. Hay, et al., submitted). Importantly, the key role of CD46 in controlling T cell differentiation is supported by the analysis of CD46-deficient patients. These patients exhibit reduced Th1 cell-mediated responses along with recurrent infections [51]. Similarly, studies using T cells from C3-deficient patients have highlighted the importance of C3 in Th1 responses. These patients exhibit impaired secretion of IFNγ and IL-10. Blockage of C3aR in healthy CD4+ T cells also resulted in diminished IFNγ secretion and IL-10 switching [73]. In both CD46- and C3-deficient patients, Th2 responses appeared to remain intact [51].

As mentioned previously, CD35 suppresses B cell activation [37]. Reduced expression of CD35 in a number of autoimmune conditions such as rheumatoid arthritis and systemic lupus erythematosus (SLE) has been obsvered, although whether these reduced levels contribute to disease remains to be firmly established [96].

Due to the high occurrence of the autoimmune condition SLE associated with C1q deficiency [97], C1q is believed to play a role in immune tolerance [98]. C1q binds directly to the apoptotic cell marker phosphatidylserine which aids in the clearance of apoptotic cells by the innate immune system [99]. C1q deficiency leads to accumulation of apoptotic cells and “blebs” containing large quantities of nuclear antigen such as dsDNA which may lead to an auto-immune response [98].

### Targeting CD46 as therapy

The analysis of the in vivo function of CD46 has been largely impaired by the lack of expression of CD46 on somatic cells in mice. Non-human primates are the only animals expressing CD46 in a similar fashion as humans. There have been a few models of CD46-transgenic mice established, and we are developing a model of CD46-expressing zebrafish that will allow the tracking of CD46-expressing T cells upon inflammation and provide a potential model to screen drugs affecting this pathway (G. Morisse, D. Sieger, A. Astier, unpublished data). Targeting of the complement regulatory function of CD46 with mutant proteins derived from adenovirus Ad35 that bind to CD46 with very high affinity enhances response to Rituximab, an anti-CD20 antibody in lymphomas in non-human primates [100]. Moreover, this mutant protein was also able to modulate the CD46 pathway in T cells, suggesting that it may be used to modulate other diseases, independently of its complement regulatory function [101]. These data underline that targeting of complement pathways may be a valid approach for future therapies.

## Conclusion and perspectives

Complement components and their interactions with their cognate receptors are therefore key in the control of adaptive immune responses. The most studied cells so far have been T cells, and as discussed above, complement regulates T cell activation, differentiation, and metabolism. This offers the perspective of using complement components as novel drug targets for chronic inflammatory diseases and autoimmune diseases. Intracellular activation of complement is now suggested not only in immune cells but also in other cells. Intriguingly, this suggests a much broader role of complement in general cell functions. There is no doubt that research in the coming years will reveal unexpected roles for complement in this regard and highlight how this ancient evolutionary system plays a much more preponderant role in the control of basic cellular mechanisms than initially thought.
